# Visualization of activity-regulated BDNF expression in the living mouse brain using non-invasive near-infrared bioluminescence imaging

**DOI:** 10.1186/s13041-020-00665-7

**Published:** 2020-09-07

**Authors:** Mamoru Fukuchi, Ryohei Saito, Shojiro Maki, Nami Hagiwara, Yumena Nakajima, Satoru Mitazaki, Hironori Izumi, Hisashi Mori

**Affiliations:** 1 Laboratory of Molecular Neuroscience, Faculty of Pharmacy, Takasaki University of Health and Welfare, 60 Nakaorui-machi, Takasaki, Gunma 370-0033 Japan; 2 Department of Engineering Science, Graduate School of Informatics and Engineering, The University of Electro-Communications, 1-5-1 Chofugaoka, Chofu, Tokyo, 182-8585 Japan; 3 School of Pharmacy, Tokyo University of Pharmacy and Life Science, 1432-1 Horinouchi, Hachioji, Tokyo, 192-0392 Japan; 4 Department of Molecular Neuroscience, Graduate School of Medicine and Pharmaceutical Sciences, University of Toyama, 2630 Sugitani, Toyama, Toyama 930-0194 Japan

**Keywords:** Bioluminescence, Brain-derived neurotrophic factor, In vivo imaging, Near-infrared

## Abstract

Altered levels of brain-derived neurotrophic factor (BDNF) have been reported in neurologically diseased human brains. Therefore, it is important to understand how the expression of BDNF is controlled under pathophysiological as well as physiological conditions. Here, we report a method to visualize changes in BDNF expression in the living mouse brain using bioluminescence imaging (BLI). We previously generated a novel transgenic mouse strain, *Bdnf-Luciferase* (*Luc*), to monitor changes in *Bdnf* expression; however, it was difficult to detect brain-derived signals in the strain using BLI with *d*-luciferin, probably because of incomplete substrate distribution and light penetration. We demonstrate that TokeOni, which uniformly distributes throughout the whole mouse body after systematic injection and produces a near-infrared bioluminescence light, was suitable for detecting signals from the brain of the *Bdnf-Luc* mouse. We clearly detected brain-derived bioluminescence signals that crossed the skin and skull after intraperitoneal injection of TokeOni. However, repeated BLI using TokeOni should be limited, because repeated injection of TokeOni on the same day reduced the bioluminescence signal, presumably by product inhibition. We successfully visualized kainic acid-induced *Bdnf* expression in the hippocampus and sensory stimulation-induced *Bdnf* expression in the visual cortex. Taken together, non-invasive near-infrared BLI using *Bdnf-Luc* mice with TokeOni allowed us to evaluate alterations in BDNF levels in the living mouse brain. This will enable better understanding of the involvement of BDNF expression in the pathogenesis and pathophysiology of neurological diseases.

## Introduction

Brain-derived neurotrophic factor (BDNF), a member of the neurotrophin family, is fundamentally involved in a variety of functions in the developing and mature brain [1]. Consistent with the crucial roles of BDNF in the central nervous system (CNS), alterations in BDNF levels have been found in the brains of patients with neurodegenerative or neuropsychiatric diseases [2–4]. Abnormal expression levels of BDNF have been reported in the postmortem brains of Alzheimer’s disease [5], Parkinson’s disease [6], Huntington’s disease [7], depression [8], and schizophrenia [9]. Higher expression levels of BDNF in the brain (dorsolateral prefrontal cortex) correlate with slower cognitive decline [10]. Furthermore, lower levels of BDNF in cerebrospinal fluids are associated with the progression of mild cognitive impairment to Alzheimer’s disease [11]. These findings indicate that a reduction of BDNF levels in the brain may trigger CNS dysfunction, resulting in neurological diseases. However, because neuronal *Bdnf* expression is regulated by neuronal activity [12], it is also plausible that neuronal dysfunction in neurological diseases can result in a reduction of BDNF levels in the brain. Despite numerous studies reporting reduced levels of BDNF in neurologically diseased brains, there is no evidence showing whether reduced BDNF levels in the brain are the cause or result of a disease.

Bioluminescence imaging (BLI) is a popular technique for monitoring changes in expression levels of target molecules. Compared to fluorescence imaging using fluorescent molecules, such as green fluorescent protein, signal intensity obtained by BLI is relatively low, and the addition of a substrate is necessary to obtain signals. However, excitation lights, which can be toxic, are not required, and the signals can be obtained non-invasively with high signal to noise ratio [13, 14]. We previously generated a novel transgenic mouse strain termed *Bdnf-Luciferase* (*Luc*) to monitor changes in *Bdnf* expression in vivo as well as in vitro, using a firefly Luc as an imaging probe [15, 16]. In this mouse strain, expression levels of Luc reflect endogenous *Bdnf* expression. Because levels of Luc can be evaluated by measuring bioluminescence produced by reaction with a substrate, such as *d*-luciferin, the most popular and commonly used substrate for in vitro and in vivo BLI, changes in *Bdnf* expression can be evaluated by detecting bioluminescence signals. The induction of *Bdnf* expression can be visualized in living neuronal cell cultures [15, 16]. In addition, bioluminescence signals from living *Bdnf-Luc* mice can be detected after intraperitoneal administration of *d*-luciferin [16]. However, despite endogenous *Bdnf* being highly expressed in the brain, signals from the brain were poorly detected in the mice [16]. The emission maximum of bioluminescence light produced by firefly Luc with *d*-luciferin is 578 nm at 25 °C and 612 nm at 37 °C [17] and, therefore, does not penetrate biological tissues well, because of light absorption by hemoglobin and melanin in the tissues [18, 19]. In addition, a heterogeneous biodistribution of *d*-luciferin has been reported [20, 21]. Furthermore, *d*-luciferin is a specific substrate for an ATP-binding cassette (ABC) transporter G2 (ABCG2) [22] and, therefore, it may limit an ability of *d*-luciferin to cross blood-brain-barrier (BBB). To improve BLI, novel substrates for Luc have been developed. For example, CycLuc1, a synthetic luciferin, has been shown to greatly improve the sensitivity of BLI, although the emission maximum is 612 nm [23]. Previously, Cao et al., (2018) reported in vivo imaging of myelination events using myelin basic protein promoter-driven Luc transgenic mice and CycLuc1 [24]. In addition, CycLuc1 amide nicely improved to detect bioluminescence signals from the brain in particular [25]. Furthermore, firefly Luc has been mutated to optimize the detection of bioluminescence from the brain using synthetic luciferins CycLuc1, CycLuc2, and their respective amides [26]. Iwano et al., (2013) developed a series of firefly Luc analogues to improve light penetration [27]. AkaLumine hydrochloride (also called TokeOni) is a novel Luc substrate that produces near-infrared light with a wavelength of approximately 680 nm and enables visualization of signals from deep tissues [28]. Furthermore, firefly Luc has been optimized for TokeOni, and an engineered BLI systems, termed AkaBLI, enables visualization of bioluminescence signals from the brain of a freely moving animal [29]. We previously detected signals from brain regions after the systematic injection of TokeOni into *Bdnf-Luc* mice; however, the signals were detected by an invasive method (we removed the skin to expose the skull before in vivo imaging) [16], and it is still unclear whether changes in *Bdnf* expression under physiological conditions can be visualized by non-invasive in vivo BLI.

In the present study, we examined the properties of two Luc substrates, seMpai and TokeOni, both of which produce near-infrared light, using *Bdnf-Luc* mice, and found that TokeOni to be the most suitable substrate for detecting bioluminescence signals from mouse brain regions non-invasively. We successfully visualized drug-induced and sensory stimulation-induced *Bdnf* expression in the living *Bdnf-Luc* mouse brain, although repeated BLI using TokeOni should be limited, presumably because of product inhibition. This report shows that induction of *Bdnf* expression in the mouse brain can be visualized under physiological conditions, and this non-invasive in vivo BLI method will facilitate further investigation of the roles of BDNF in neurological disease. In addition, this report provides instructive information for the in vivo use of TokeOni with other Luc mice line.

## Methods

### Animals

All animal care procedures and experiments were approved by the Animal Experiment Committee of the University of Toyama (Authorization No. S-2010 MED-51, A2011PHA-18, and A2014PHA-1) and Takasaki University of Health and Welfare (Authorization No. 1733, 1809, 1913, and 2008), and were performed in accordance with the Guidelines for the Care and Use of Laboratory Animals of the University of Toyama and Takasaki University of Health and Welfare. Mice were housed under standard laboratory conditions (12 h–12 h/light-dark cycle at 22 ± 2 °C) and had free access to food and water. The generation of *Bdnf-Luc* mice has been described previously [15, 16] and 8–14 week-old *Bdnf-Luc* mice were used.

### In vivo BLI

One day before in vivo BLI, the black fur was shaved from the top of the head of *Bdnf-Luc* mice under inhalation anesthesia with 2.0% isoflurane. *d*-luciferin (Promega, Madison, WI, USA), TokeOni, and seMpai were dissolved in saline at the concentration of 10 mg/ml. *Bdnf-Luc* mice were anesthetized by inhalation of 2.0% isoflurane, and then Luc substrate solution was administered intraperitoneally [0.1 ml substrate solution/10 g body weight (dose of each substrate: 100 mg/kg)]. In our previous report, TokeOni was used at 150 mg/kg or 75 mg/kg, and the signals from the brain region were successfully detected [16]. Therefore, in the current study, we determined the dose of TokeOni at 100 mg/kg. To compare the bioluminescence signals in the same conditions, the dose of the other substrates was also determined at 100 mg/kg. Five minutes after substrate administration, BLI was performed using an IVIS in vivo imaging system [PerkinElmer, Boston, MA, USA (Exposure time: 2 min, Binning: Medium, F/Stop: 1)]. Pseudocolored bioluminescent images representing the spatial distribution of emitted photons were overlaid on photographs of the mouse taken in the chamber. The results shown in Supplementary Figure 2 were generated by in vivo BLI performed according to our previous report [16].

### KA administration and analysis of endogenous BDNF expression

Kainic acid [KA (Sigma-Aldrich, St. Louis, MO, USA)] was dissolved in saline at 2.5 mg/ml. Saline or KA solution was administered intraperitoneally to *Bdnf-Luc* mice [0.1 ml substrate solution/10 g body weight (dose of KA: 25 mg/kg)]. Six hours after the administration of saline or KA, in vivo BLI was performed using TokeOni. After BLI, the mice were decapitated while still anesthetized and cerebral cortex and hippocampus were isolated to examine changes in endogenous *Bdnf* mRNA and BDNF protein levels.

Total RNA was purified from the cerebral cortex and hippocampus using ISOGEN (Nippongene, Tokyo, Japan), according to the manufacturer’s instructions. One microgram of purified total RNA was reverse-transcribed into cDNA using a PrimeScript 1st Strand cDNA Synthesis Kit (TaKaRa Bio, Kusatsu, Japan), according to the manufacturer’s instructions. Real-time PCR was performed using SYBR Select Master Mix (Thermo Fisher Scientific, Waltham, MA, USA), according to the manufacturer’s instructions. Fold-change values were calculated by the ^ΔΔ^Ct method to determine relative gene expression. Primer sequences of *Bdnf* and *Gapdh* were as described previously [16]. The levels of *Bdnf* mRNA were normalized to those of *Gapdh* mRNA.

Protein extraction was performed using T-PER Protein Extraction Reagent (Thermo Fisher Scientific) supplemented with Halt Protease Inhibitor Cocktail (Thermo Fisher Scientific), according to the manufacturer’s instructions. Protein concentrations were determined using a BCA Protein Assay Kit (Thermo Fisher Scientific). After heat denaturation of samples in Laemmli Sample Buffer (BioRad, Hercules, CA, USA) supplemented with 2-mercaptoethanol, 10 μg of protein was separated by SDS-PAGE (for BDNF: 15% polyacrylamide gel, for α-Tubulin: 10% polyacrylamide gel). Separated proteins were transferred to a PVDF membrane. The membrane was washed, blocked with 5% skimmed milk, and then treated with a primary antibody {anti-BDNF antibody [Abcam, Cambridge, UK (ab108319, 1:1000)] or anti-α-Tubulin antibody [Wako, Osaka, Japan (1:1000)]} diluted in Can Get Signal Solution 1 (TOYOBO, Osaka, Japan) overnight at 4 °C with shaking. The membrane was washed, treated with a secondary antibody {anti-rabbit IgG HRP-conjugated [GE Healthcare, Buckinghamshire, England (1:5000)] or anti-mouse IgG HRP-conjugated [GE Healthcare, (1:5000)]} diluted in Can Get Signal Solution 2 (TOYOBO) for 1 h at room temperature with shaking, and then washed. Each band was detected using ImmunoStar Zeta (Wako). Intensity of each band was measured using Image J. The levels of BDNF were normalized to those of α-Tubulin.

### Sensory stimulation

The black fur was shaved from the top of the head of *Bdnf-Luc* mice under inhalation anesthesia with 2.0% isoflurane, and then the mice were housed in the dark for 6.5 days. We then performed in vivo BLI using TokeOni without lighting. After BLI, the mice were housed in the dark for a further 2 days, and then the mice were exposed to light for 1 h. After light exposure, the mice were housed in the dark for 5 h, and then in vivo BLI was performed again. Region of interest (ROI) analysis was performed according to previous reports [30, 31] with modifications. Briefly, the region of the cerebral cortex was estimated by the bioluminescence signal image (Supplementary Fig. 3a, the region surrounded by a red line), and the region was covered with 16 × 24 ROIs (Supplementary Fig. 3a, 16 × 24 boxes shown in white line). ROIs containing visual cortex (ROI V1 and V2) or somatosensory cortex (ROI S1 and S2) were estimated by mouse brain atlas.

### Statistics

All data are presented as the mean ± the standard error of the mean (SEM). Statistical analyses were performed using Prism 7 software (GraphPad). Detailed information regarding statistical analysis of each result is shown in each figure legend.

## Results

### Detection of bioluminescence signals from the living *Bdnf-Luc* mouse brain

**Fig. 1:**
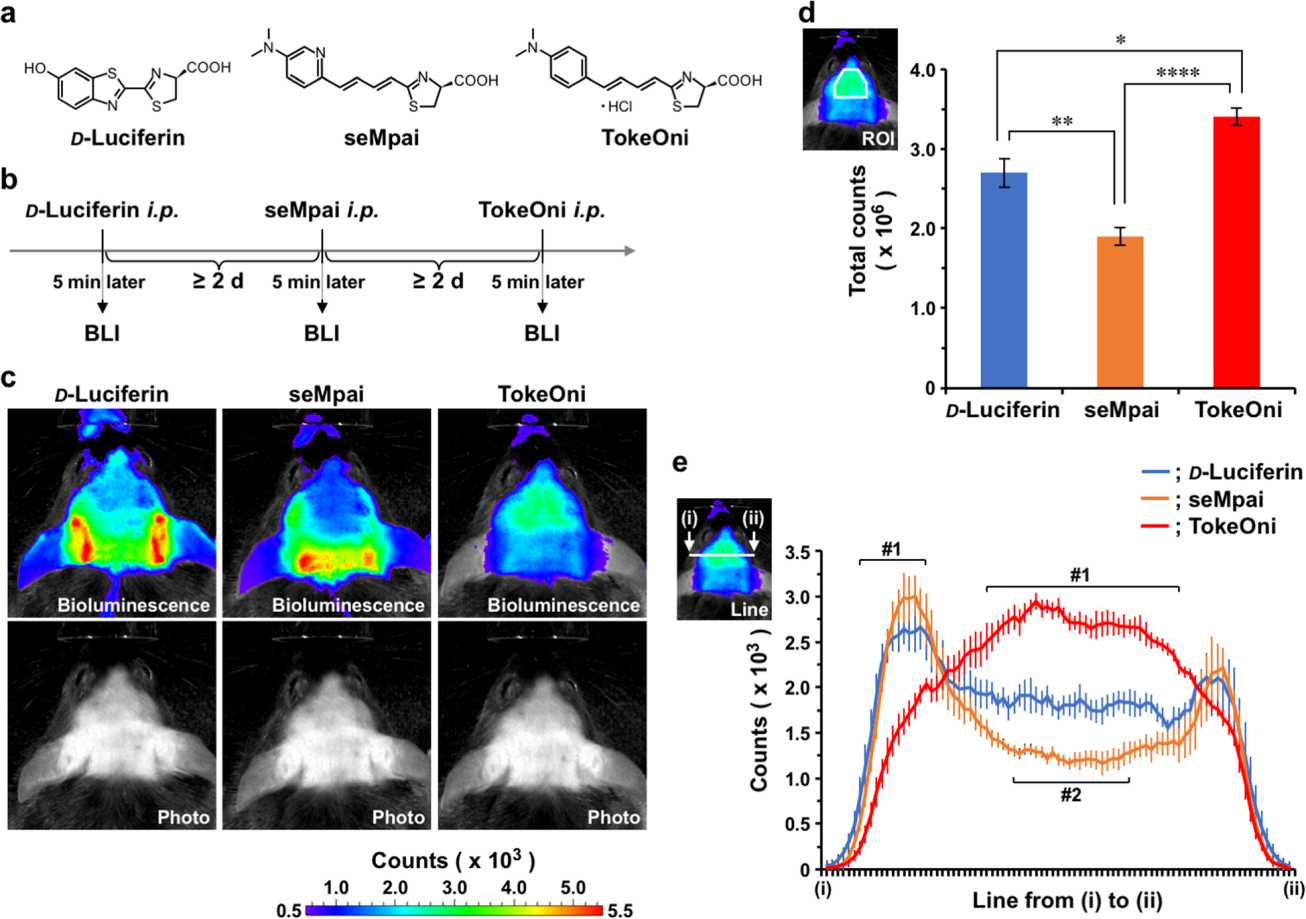
Comparison of luciferase substrates for in vivo BLI using *Bdnf-Luc* mice. **a.** Structure of *d*-luciferin, seMpai, and TokeOni. **b.** Schedule of experiments. *d*-luciferin, seMpai, and TokeOni were administered intraperitoneally to *Bdnf-Luc* mice, and in vivo BLI was performed 5 min after each administration. Each substrate was injected into mice with at least 2 d intervals. **c.** Representative images of in vivo BLI using *d*-luciferin, seMpai, or TokeOni as a luciferase substrate. Bioluminescence; counts indicated by pseudocolored images. Photo; photographs corresponding to bioluminescence images. **d.** ROI analysis. Data represent the mean ± SEM of four independent experiments using one-way ANOVA with Tukey’s multiple comparisons test (*p < 0.05, **p < 0.01, and ****p < 0.0001). **e.** Line profiles [counts from (i) to (ii)]. Data represent the mean ± SEM of four independent experiments using two-way ANOVA with Dunnett’s multiple comparisons test [#1; significant difference between *d*-Luciferin versus TokeOni (*p* < 0.05), #2; significant difference between *d*-Luciferin versus seMpai (*p* < 0.05)]

We first tried to identify a suitable substrate of firefly Luc to enable visualization of changes in *Bdnf* expression in living *Bdnf-Luc* mouse brains using non-invasive in vivo BLI. We used *d*-luciferin, TokeOni, and seMpai, as Luc substrates (Fig. 1a). TokeOni and seMpai are synthetic luciferins and produce near-infrared light [27, 28, 32]. TokeOni barely dissolves in a neutral pH buffer; a solution with an acidic pH is required, which may be unsuitable for certain experiments. In contrast, seMpai can be dissolved in neutral pH solvents.

To compare the detection of bioluminescence signals produced by each substrate, we administered each substrate to *Bdnf-Luc* mice under inhalation anesthesia and then measured bioluminescence signals (Fig. 1b). Endogenous BDNF is highly abundant in the brain; therefore, strong bioluminescence signals were expected from the brain. However, we could not identify the region of cerebral cortex after intraperitoneal injection of *d*-luciferin (Fig. 1c). On the other hand, we detected signals from the brain after intracerebroventricular injection of *d*-luciferin (Supplementary Fig. 1). The signal intensity from the head region obtained using seMpai as a Luc substrate was lower compared with that obtained using *d*-luciferin (Fig. 1c). In contrast, we clearly detected signals from brain regions, probable the region of cerebral cortex in particular, after injection of TokeOni (Fig. 1c). Both ROI analysis (Fig. 1d) and line profiles (Fig. 1e) showed that higher signal intensities from brain regions were detected using TokeOni compared with using *d*-luciferin and seMpai. Furthermore, in previous experiments we removed the skin to expose the skull before in vivo BLI [16]; however, bioluminescence signals were clearly detected from the brain of *Bdnf-Luc* mice using TokeOni (Fig. 1c–e), indicating that it is not necessary to expose the skull before in vivo BLI. Thus, TokeOni was the most suitable substrate tested for the non-invasive visualization of BDNF expression levels in the living *Bdnf-Luc* mouse brain.

### Visualization of kainic acid-induced *Bdnf* expression in living mouse hippocampus

**Fig. 2:**
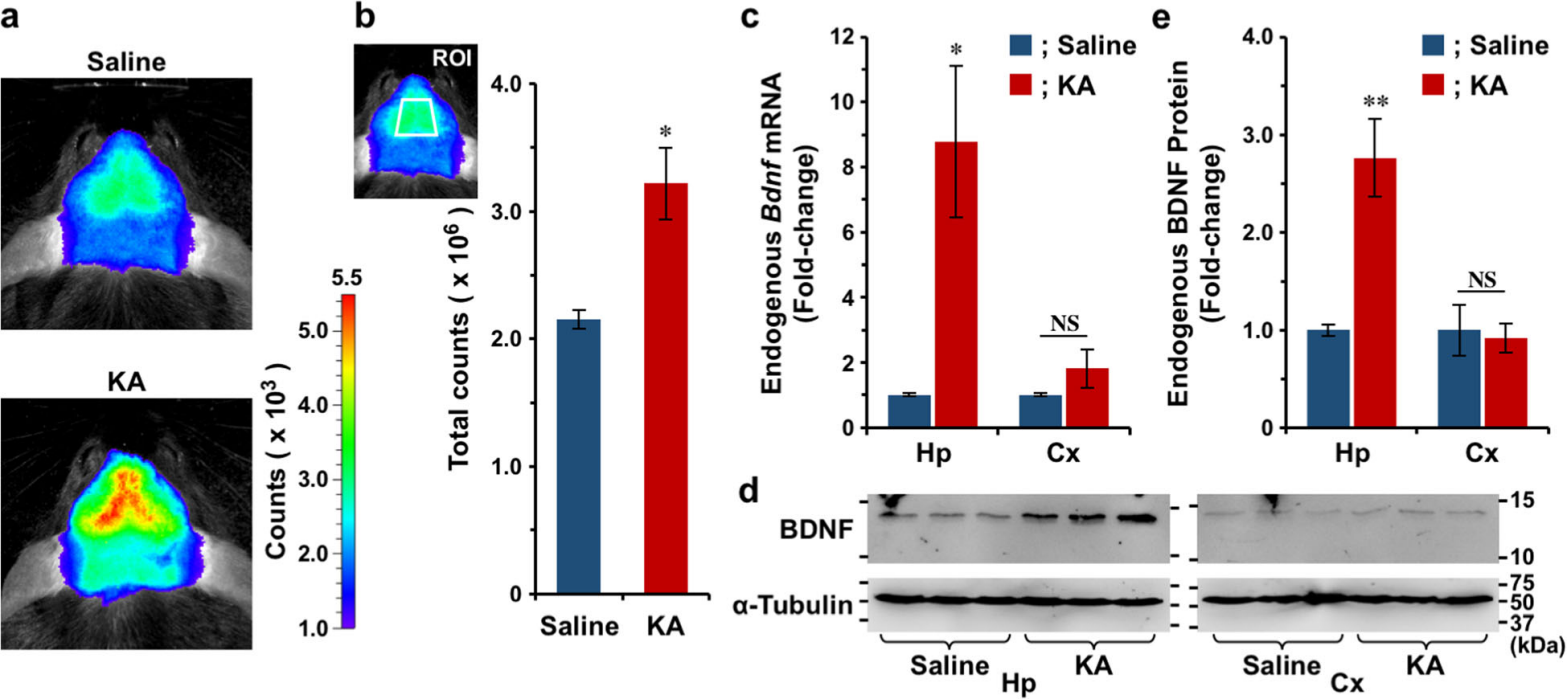
Visualization of KA-induced *Bdnf* expression. **a.** Representative images of in vivo BLI 6 h after administration of saline or KA. **b.** ROI analysis. Data represent the mean ± SEM of three independent experiments using the unpaired *t* test (*p < 0.05). **c.** RT-PCR analysis. After in vivo BLI, total RNA was prepared from the hippocampus (Hp) and cerebral cortex (Cx) of *Bdnf-Luc* mice to examine changes in endogenous *Bdnf* mRNA levels. Data represent the mean ± SEM of three independent experiments using the unpaired *t* test (*p < 0.05, NS; not significant). **d.** Immunoblot analysis. After in vivo BLI, proteins were extracted from the hippocampus and cerebral cortex of *Bdnf-Luc* mice to examine changes in endogenous BDNF protein levels. **e.** The intensities of bands shown in Fig. 2d were quantified using Image J. Data represent the mean ± SEM of three independent experiments using the unpaired *t* test (**p < 0.01, NS; not significant)

In our previous study, we successfully visualized the induction of *Bdnf* expression after intracerebroventricular injection of pituitary adenylate cyclase-activating polypeptide [16], which increases *Bdnf* expression in the cerebral cortex [15]. In this study, we examined whether the induction of *Bdnf* expression could be visualized non-invasively in *Bdnf-Luc* mice by in vivo BLI using TokeOni. Kainic acid (KA) increases *Bdnf* expression in the rodent brain [33, 34]; however, we could not detect significant changes in bioluminescence signals after KA administration to *Bdnf-Luc* mice when we use *d*-luciferin as a Luc substrate (Supplementary Fig. 2). Here, we administered saline or KA to *Bdnf-Luc* mice and then measured bioluminescence signals using TokeOni as a Luc substrate. Compared with signals from saline-administered mice, the signals from the brain were clearly increased after KA administration (Fig. 2a). ROI analysis revealed that the signals from the brain were significantly increased by KA administration (Fig. 2b). In addition, the signals seemed to be strongly increased in the hippocampus (Fig. 2a). To confirm this, we investigated the expression levels of endogenous BDNF in the hippocampus and cerebral cortex after in vivo BLI. Both *Bdnf* mRNA (Fig. 2c) and BDNF protein (Fig. 2d, e) levels were significantly increased by KA administration in the hippocampus but not in the cerebral cortex of *Bdnf-Luc* mice. These results strongly indicated that changes in endogenous *Bdnf* expression could be visualized in the living *Bdnf-Luc* mouse brain by in vivo BLI with TokeOni.

### Limitation of using TokeOni for repeated in vivo BLI

**Fig. 3:**
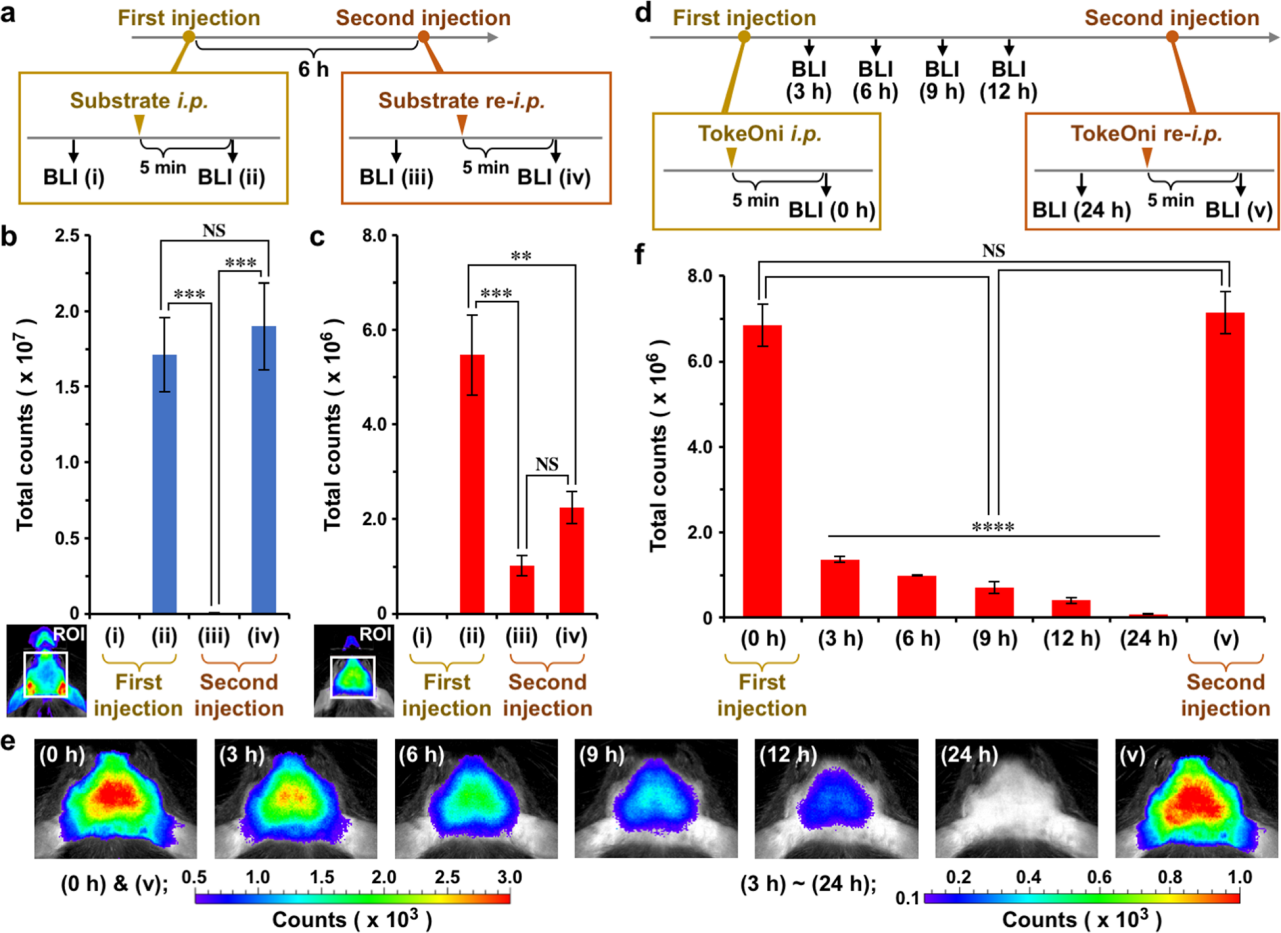
Limitation of using TokeOni for repeated in vivo BLI. **a.** Schedule of experiments. In vivo BLI was performed before (i) and after (ii) the intraperitoneal administration of each substrate to *Bdnf-Luc* mice (First injection). Six hours after the first injection, in vivo BLI was performed before (iii) and after (iv) substrate administration again (Second injection). **b.** ROI analysis to measure counts at each step. *d*-luciferin was administered according to the schedule shown in Fig. 3a. Data represent the mean ± SEM of three independent experiments using one-way ANOVA with Tukey’s multiple comparisons test (***p < 0.001, NS; not significant). **c.** ROI analysis. TokeOni administration and in vivo BLI were performed according to the schedule shown in Fig. 3a. Data represent the mean ± SEM of four independent experiments using one-way ANOVA with Tukey’s multiple comparisons test (**p < 0.01, ***p < 0.001, NS; not significant). **d.** Schedule of experiments. In vivo BLI was performed 5 min after the intraperitoneal administration of TokeOni to *Bdnf-Luc* mice (0 h). Then, in vivo BLI was repeatedly performed at 3, 6, 9, 12, and 24 h without further TokeOni administration. Twenty-four hours after in vivo BLI, TokeOni was re-injected into the mice, and in vivo BLI was performed again (v). **e.** Representative images of in vivo BLI. The pseudocolored range shown to the left (from 0.5 × 10^3^ to 3.0 × 10^3^) corresponds to the images at (0 h) and (v), and the range shown to the right (from 0.2 × 10^3^ to 1.0 × 10^3^) corresponds to the other images. **f.** ROI analysis. TokeOni administration and in vivo BLI were performed according to the schedule shown in Fig. 3d. Data represent the mean ± SEM of four independent experiments using one-way ANOVA with Tukey’s multiple comparisons test (****p < 0.0001, NS; not significant)

One of the advantages of non-invasive BLI to evaluate changes in target gene expression is repeated measurements in the same individual. However, it is necessary to administer a Luc substrate at each measurement. Therefore, we next examined whether TokeOni could be repeatedly administered to *Bdnf-Luc* mice. Five minutes after the administration of *d*-luciferin to mice, we could detect bioluminescence signals (Fig. 3a, b). The signals were barely detectable 6 h after the administration but could be detected again by re-administration of *d*-luciferin (Fig. 3a, b). The signal intensity after the second injection was almost the same as the intensity after the first injection (Fig. 3b), indicating that *d*-luciferin can be repeatedly used for in vivo BLI. Compared with signals detected 5 min after the administration of TokeOni to mice, the signal strength was decreased but still detectable 6 h after the administration (Fig. 3a, c). However, the signal intensity after the second injection was significantly lower than the intensity after the first injection (Fig. 3c). To examine this response further, we administered TokeOni to *Bdnf-Luc* mice once and then performed in vivo BLI at 0, 3, 6, 9, 12, and 24 h after the administration (Fig. 3d). Compared to signals at 0 h, the signals were reduced but detectable 3 h after the administration (Fig. 3e, f). The signals were still detectable at 12 h, but very weak at 24 h after the administration (Fig. 3e, f). Twenty-four hours after the first injection, we re-administered TokeOni to the mice and could detect signals at comparable levels to those after the first injection (Fig. 3e, f). Thus, although TokeOni is a beneficial substrate for detecting bioluminescence signals from living mouse brains non-invasively, the substrate should be administered to mice at appropriate intervals, such as once a day.

### Visualization of sensory-driven *Bdnf* expression in the living mouse visual cortex

**Fig. 4:**
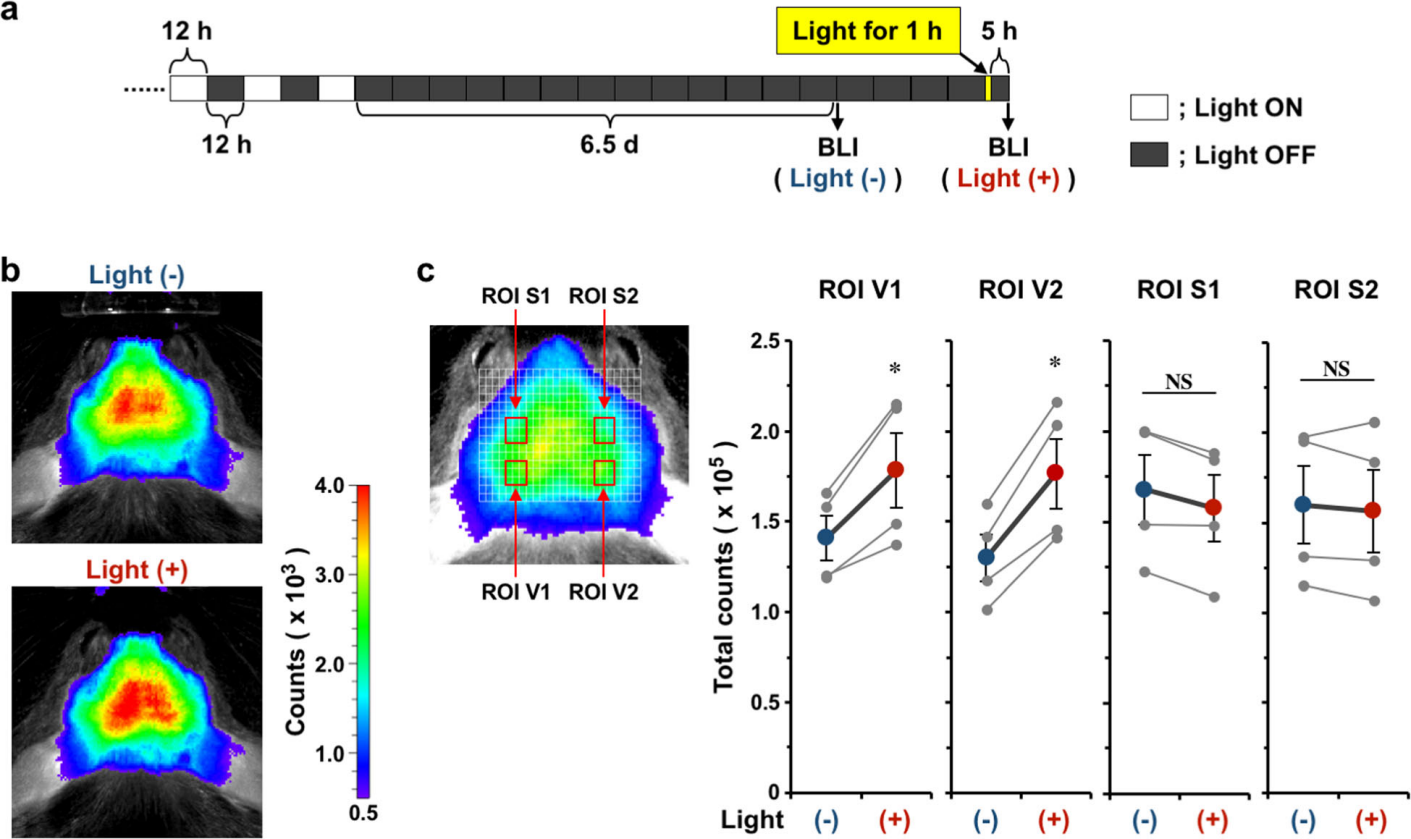
Visualization of sensory stimulation-induced *Bdnf* expression. **a.** Schedule of experiments (also refer to the Materials and methods). **b.** Representative images of in vivo BLI. In vivo BLI was performed 6.5 d after houseing *Bdnf-Luc* mice in the dark [Light (−)]. The mice were housed in the dark for an additional 2 d, and were then exposed to light for 1 h. After light exposure for 1 h, the mice were again housed in the dark for 5 h, and then in vivo BLI was performed [Light (+)]. **c.** ROI analysis. ROI V1 and V2 contains the visual cortex, and ROI S1 and S2 contains the somatosensory cortex (also refer to Supplementary Fig. 3 in detail). Data represent the mean ± SEM of four independent experiments using the paired *t* test (*p < 0.05, NS; not significant)

We next tried to visualize the induction of *Bdnf* expression in the living *Bdnf-Luc* mouse brain under physiological conditions. Light exposure increases BDNF expression in the visual cortex [35, 36]. We, therefore, housed *Bdnf-Luc* mice in the dark for 6.5 days and then performed BLI [Fig. 4a, Light (−)]. After BLI, the mice were again housed in the dark. Two days after the first BLI, the mice were exposed to light for 1 h, housed in the dark for 5 h, and BLI signals measured again [Fig. 4a, Light (+)]. Compared with the signals from the brain of *Bdnf-Luc* mice housed in the dark, light exposure for 1 h increased the signal intensity (Fig. 4b). The signals were likely to be higher in the visual cortex; therefore, we performed ROI analysis (Supplementary Fig. 3). The signals in ROI V1 and V2, the region containing the visual cortex, were significantly increased after light exposure (Fig. 4c). In contrast, the signals in ROI S1 and S2, the region containing the somatosensory cortex, did not change in response to light (Fig. 4c). Thus, we successfully visualized the induction of *Bdnf* expression in the visual cortex of living *Bdnf-Luc* mice in response to sensory stimulation.

## Discussion

We previously generated a transgenic mouse strain, *Bdnf-Luc*, to visualize changes in *Bdnf* expression in living cells and mice [15, 16]. However, *d*-luciferin was not suitable for visualizing changes in *Bdnf* expression in the living mouse brain. One of the problems regarding the detection of bioluminescence signals produced by *d*-luciferin in *Bdnf-Luc* mouse brain was the poor ability of the substrate to cross the BBB [20, 21]. The ability of *d*-luciferin to cross the BBB may be limited by ACBG2 [22]. This is also supported the detection of signals from the brain when *d*-luciferin was injected directly into the brain ventricles of *Bdnf-Luc* mice. Furthermore, the signals obtained from the head region using seMpai were lower than those produced by *d*-luciferin, suggesting that seMpai may be less able to cross the BBB compared with *d*-luciferin. The signals produced by *d*-luciferin, as well as seMpai, were also detected in the regions without black fur. These signals were probably derived from surface tissues such as skin, as previously reported [16]. We confirmed that endogenous *Bdnf* mRNA was expressed in the skin of the head region [16]. However, the signals were strongly detected in the base of the ears in particular, when we injected *d*-luciferin to the mice. Further investigations are necessary to identify the bioluminescence signals from peripheral tissues. A hairless mouse strain [31] would help us further examine peripheral *Bdnf* expression by in vivo BLI. In contrast, TokeOni produced signals in the brain, reflecting the high expression levels of endogenous BDNF in the brain.

The other problem of bioluminescence tissue penetration was also solved by using TokeOni, because it produces near-infrared bioluminescence. In our previous study, we removed the skin from the top of the skull of *Bdnf-Luc* mice before in vivo BLI, even if TokeOni was used [16]. However, in this study, we found that the signals were detectable after crossing the skull and skin. In addition, our current results regarding KA-induced *Bdnf* expression demonstrated that the signals in the hippocampus could be detected. Because previous reports suggest that KA disrupts the BBB [37], it might be possible that KA-induced increase in the bioluminescence signals is due to the BBB dysfunction. If so, the KA-induced signals would be also observed using *d*-luciferin. However, we could not observe the significant changes in the signals after KA administration when we used *d*-luciferin as a substrate for Luc. On the other hand, it has been shown that the signals obtained by TokeOni have also been detected from the striatum [29]. Thus, we suggest that TokeOni will enable non-invasive in vivo BLI and also bioluminescence signals from deeper brain regions to be detected. In the previous report, bioluminescence signals were successfully detected in freely moving animals using AkaBLI [29]. Therefore, it would be possible to visualize changes in *Bdnf* expression, if the firefly Luc in *Bdnf-Luc* mice is replaced by Akaluc, which is an optimized firefly Luc for TokeOni.

Our current results may reflect differences in the pharmacokinetics of *d*-luciferin and TokeOni in mice. The in vitro *K*m value of TokeOni is lower than that of *d*-Luciferin [28], suggesting that the affinity of TokeOni to Luc is higher than that of *d*-luciferin, which would result in the long-lasting detection of signals produced by TokeOni in vivo. Furthermore, the second signals were significantly reduced when TokeOni was administered to *Bdnf-Luc* mice at 6 h intervals. This reduction is probably caused by product inhibition [38, 39]; enzymatic reaction products of TokeOni may inhibit the Luc-TokeOni enzymatic reaction. In any case, the first and second signal intensities were comparable when TokeOni was administered to mice at 24 h intervals. Therefore, an appropriate interval of administration should be examined before TokeOni is applied to each Luc mouse line. In addition, we previously reported that luciferase activity was stably detected in primary neuronal cells prepared from *Bdnf-Luc* mice after pharmacological inhibition of de novo transcription, despite *Luc* and endogenous *Bdnf* mRNA levels being similarly decreased under the same conditions [40]. Therefore, it should be noted that rapid decreases in *Bdnf* expression and oscillatory changes in *Bdnf* expression are difficult to visualize by in vivo BLI using *Bdnf-Luc* mice.

A number of reports show lower BDNF levels in brains with neurological diseases [2–4, 10, 11]. Non-invasive near-infrared in vivo BLI using *Bdnf-Luc* mice and TokeOni will allow changes in *Bdnf* expression in the brain under physiological and pathophysiological conditions to be examined. Therefore, this method will facilitate further understanding of the relationship between alterations in BDNF levels in the brain and pathophysiology of neurological diseases, assuming that disease model mice can be generated using *Bdnf-Luc* mice. In addition, near-infrared BLI enable the detection of bioluminescence from deep tissue regions, including those of the brain. TokeOni is now commercially available; therefore, our findings also provide instructive information for the application of this substrate to other Luc mouse line.

## Supplementary information

**Additional file 1: Figure S1.** In vivo BLI after intracerebroventricular injection of *D-*luciferin into *Bdnf-Luc* mice. **Figure S2.** Detection of bioluminescence signals using *D*-luciferin 6 h after administration of saline or KA to *Bdnf-Luc* mice. **Figure S3.** Visualization of sensory stimulation-induced *Bdnf* expression (ROI analysis).

## Data Availability

All data needed to evaluate the conclusions in the paper are present in the paper.

## Abbreviations

ABCG2: ATP-binding cassette (ABC) transporter G2
BBB: Blood-brain-barrier
BDNF: Brain-derived neurotrophic factor
BLI: Bioluminescence imaging
CNS: Central nervous system
KA: Kainic acid
Luc: Luciferase
ROI: Region of interest
