# Supplementary material for: Visualization of activity-regulated BDNF expression in the living mouse brain using non-invasive near-infrared bioluminescence imaging
Source: Mol Brain. 2020 Sep 7;13:122. doi: 10.1186/s13041-020-00665-7 (PMC7487487; doi:10.1186/s13041-020-00665-7)
Supplement: Supplementary file 1 — Additional file 1: Figure S1. In vivo BLI after intracerebroventricular injection of D-luciferin into Bdnf-Luc mice. Figure S2. Detection of bioluminescence signals using D-luciferin 6 h after administration of saline or KA to Bdnf-Luc mice. Figure S3. Visualization of sensory stimulation-induced Bdnf expression (ROI analysis). [file 13041_2020_665_MOESM1_ESM.pdf]

Supplementary information:

Visualization of activity-regulated BDNF expression in the living mouse brain using non-invasive near-infrared bioluminescence imaging

Mamoru Fukuchi[1,*], Ryohei Saito[2,3], Shojiro Maki[2], Nami Hagiwara[1], Yumena Nakajima[1], Satoru Mitazaki[1], Hironori Izumi[4], Hisashi Mori[4]

Mamoru Fukuchi, fukuchi@takasaki-u.ac.jp

Ryohei Saito, rsaito@toyaku.ac.jp

Shojiro Maki, s-maki@uec.ac.jp

Nami Hagiwara, 8.8forknight@gmail.com

Yumena Nakajima, dream.smiley2@gmail.com

Satoru Mitazaki, mitazaki@takasaki-u.ac.jp

Hironori Izumi, izuchan@med.u-toyama.ac.jp

Hisashi Mori, hmori@med.u-toyama.ac.jp

[1]Laboratory of Molecular Neuroscience, Faculty of Pharmacy, Takasaki University of Health and Welfare, 60 Nakaorui-machi, Takasaki, Gunma 370-0033, Japan

[2]Department of Engineering Science, Graduate School of Informatics and Engineering, The University of Electro-Communications, 1-5-1 Chofugaoka, Chofu, Tokyo 182-8585, Japan

[3]School of Pharmacy, Tokyo University of Pharmacy and Life Science, 1432-1 Horinouchi, Hachioji, Tokyo 192-0392, Japan

[4]Department of Molecular Neuroscience, Graduate School of Medicine and Pharmaceutical Sciences, University of Toyama, 2630 Sugitani, Toyama, Toyama 930-0194, Japan

[*]To whom correspondence should be addressed: Mamoru Fukuchi, Laboratory of Molecular Neuroscience, Faculty of Pharmacy, Takasaki University of Health and Welfare, 60 Nakaorui-machi, Takasaki, Gunma 370-0033, Japan. E-mail address: fukuchi@takasaki-u.ac.jp, telephone: +81-27-352-1180, fax: +81-27-352-1118.

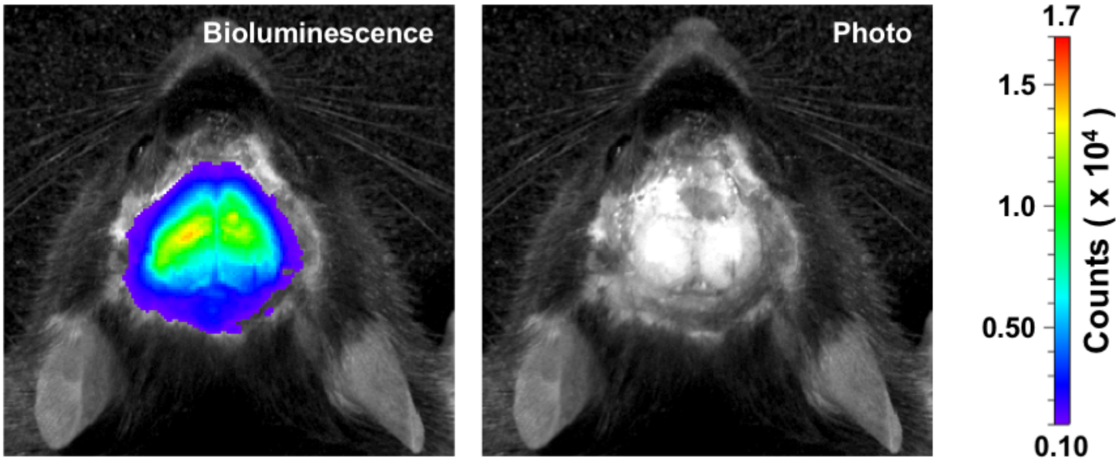

Supplementary Figure 1. ***In vivo*** **BLI after intracerebroventricular injection of D-luciferin into** ***Bdnf-Luc*** **mice.**

Bdnf-Luc mice were anesthetized with mixed anesthetic agents [medetomidine hydrochloride (0.3 mg/kg), midazolam (4 mg/kg), and butorphanol tartrate (5 mg/kg)], and the skin on the top of the head was removed. Five microliters of D-luciferin solution (10 mg/mL) was injected intracerebroventricularly (−0.2 mm anterioposterior; +1.0 mm mediolateral; −2.5 mm dorsoventral) at a rate of 1 μL/min using a microsyringe pump. The injection was performed under stereotaxic guidance. After the injection, *in vivo* BLI was performed. Representative images are shown and similar results were obtained from three independent experiments. Bioluminescence; counts indicated by pseudocolored images. Photo; photographs corresponding to bioluminescence images.

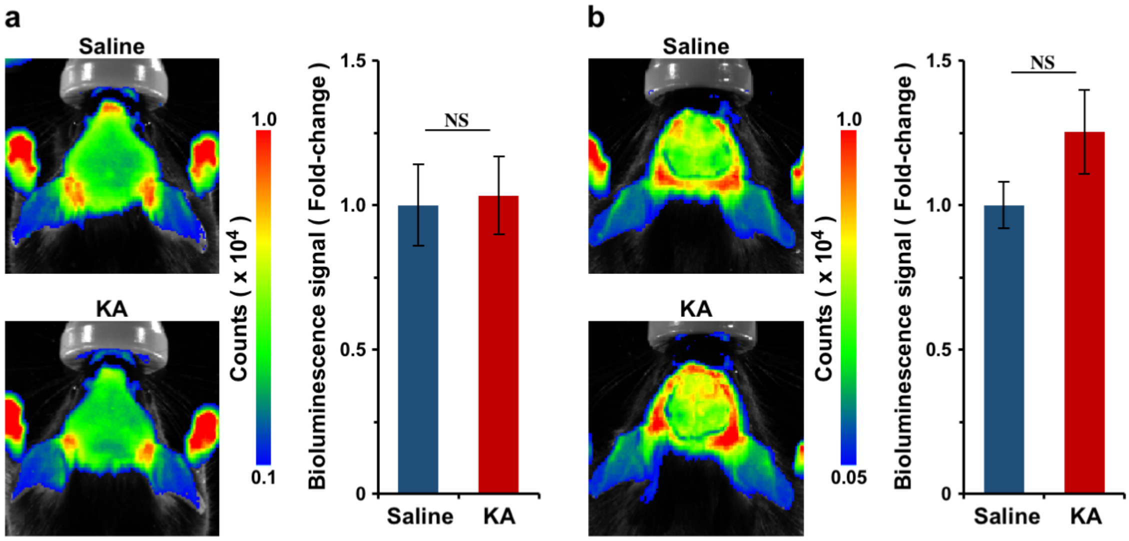

Supplementary Figure 2. Detection of bioluminescence signals using D-luciferin 6 h after administration of saline or KA to *Bdnf-Luc* mice.

These experiments were performed according to our previous report [16]. Briefly, saline or KA (25 mg/kg) was administered intraperitoneally to *Bdnf-Luc* mice. Six hours after the administration of saline or KA, D-luciferin (150 mg/kg) was injected intraperitoneally, and 10 min later, *in vivo* BLI was performed under inhalation anesthesia with 1.5% isoflurane. Data represent the mean ± SEM of six independent experiments using the unpaired *t* test (NS; not significant). **a.** One day before *in vivo* BLI, the top of the head was shaved. **b.** One week before *in vivo* BLI, the skin of the head region was removed.

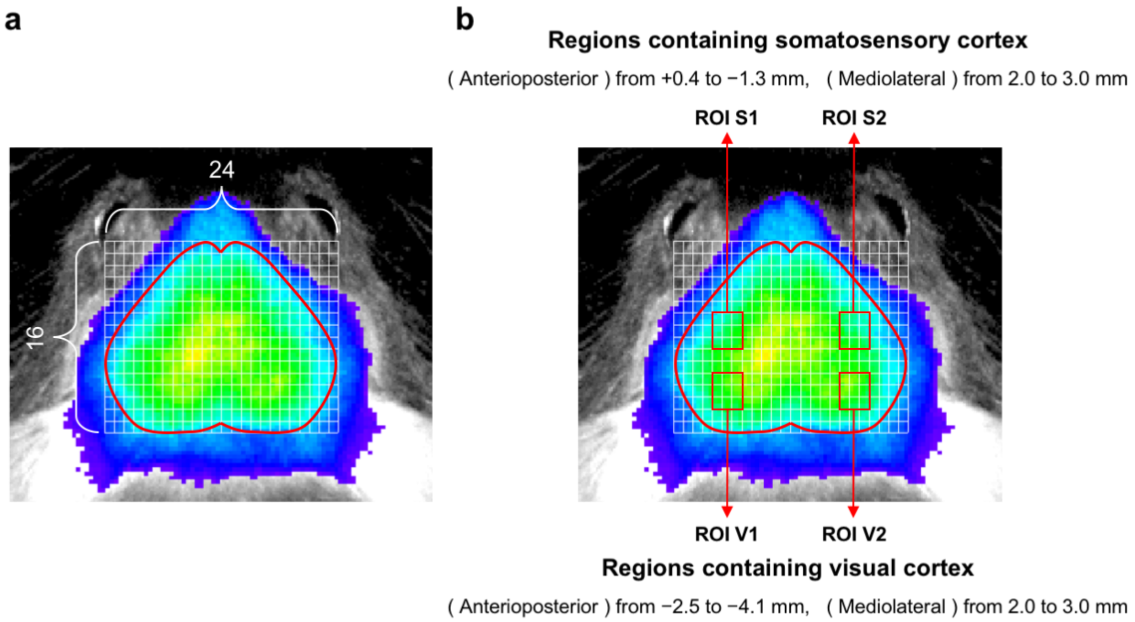

Supplementary Figure 3. Visualization of sensory stimulation-induced *Bdnf* expression (ROI analysis).

a. The area of the cerebral cortex was estimated by the bioluminescence signal image (the region surrounded by a red line), and the region was covered with 16 × 24 ROIs (16 × 24 boxes shown in white line). **b**. ROIs containing visual cortex (ROI V1 and V2) or somatosensory cortex (ROI S1 and S2) were estimated by mouse brain atlas [ROI V1 and V2 correspond to the areas from approximately −2.5 mm to −4.1 mm (anterioposterior) and from approximately 2.0 mm to 3.0 mm (mediolateral). ROI S1 and S2 correspond to the areas from approximately +0.4 mm to −1.3 mm (anterioposterior) and from approximately 2.0 mm to 3.0 mm (mediolateral)].
